# On the relationship of *online* and *offline* social cognition

**DOI:** 10.3389/fnhum.2014.00278

**Published:** 2014-05-06

**Authors:** Leonhard Schilbach

**Affiliations:** Department of Psychiatry, University Hospital CologneCologne, Germany

**Keywords:** social interaction, social neuroscience, social cognition from an observer’s point of view, social cognition from an interactor’s point of view, developmental psychology

## Abstract

Social neuroscience studies the neurobiological underpinnings of people making sense of people. Due to both conceptual and methodological constraints, the majority of studies in this field of research, however, has employed experimental paradigms that focus on *social cognition from an observer’s* rather than *from an interactor’s point of view* (*offline* vs. *online* social cognition). This calls for an increased effort to systematically investigate the neural bases of participation in real-time social interaction. In light of the ontogenetic primacy of social interaction over observation and the idea that neural networks established during social interaction may be “re-used” during observation, other important objectives of the field will be to relate new findings into the neural bases of social interaction to previous work investigating the neural bases of social observation as well as to find ways to directly compare the two.

## Social neuroscience: achievements and challenges

Social neuroscience has come a long way since its inception (Frith and Frith, 1999; Ochsner and Lieberman, 2001) and has helped to characterize the neurobiological bases of various aspects of social cognition. In particular the advent, refinement and more wide-spread use of functional magnetic resonance imaging (fMRI) has furthered insight into mapping social cognitive capacities onto brain areas and neural networks. In the process, two large-scale neural networks have taken center stage as the neurobiological substrate of social cognition: the so-called “mirror neuron system” (MNS) and the so-called “mentalizing network” (MENT). The MNS has been taken as evidence for a first-person or *simulationist* account of social cognition. By activating brain areas that would be necessary to exhibit motor behavior onself, the MNS is believed to provide an implicit, first-person grasp of others’ behavior (Caspers et al., 2010; Rizzolatti and Sinigaglia, 2010). The MENT, on the other hand, has often been taken as evidence for a third-person or *theorist* account of social cognition. It is believed by many to provide inferences with regard to the mental states that govern observed behavior and thereby help to explain and predict behavior (e.g., Frith and Frith, 2010). The apparent disparity in these research results can be traced back to both conceptual and methodological constraints, which have affected and shaped the experimental paradigms that have most often been used (cf. Keysers and Gazzola, 2007; Schilbach, 2010; Pfeiffer et al., 2013a; Schilbach et al., 2013). In light of the fact that both simulationist and theorist accounts have been described as spectator theories of social cognition, which are committed to an observer epistemology according to which knowing others is limited to perceiving them, it comes as no surprise that the neuroimaging paradigms used to provide empirical evidence ask subjects to observe others and/or think about their mental states rather than engage in direct interaction with them. In other words the employed experimental paradigms have focused on social cognition *from an observer’s* (i.e., *offline* social cognition) rather than *from an interactor’s point of view* (i.e., *online* social cognition; Schilbach et al., 2012, 2013). Consequently, it remains unclear (i) whether and how neural activity and connectivity in these canonical networks is modulated by the degree of participation in an ongoing interaction and (ii) whether the networks subserve complementary or mutually exclusive roles (Schilbach, 2010; Schilbach et al., 2013).

Until today, the neural mechanisms underlying actual social interactions in real life, therefore, are insufficiently understood. Importantly, this is not only problematic for fundamental social neuroscience research itself. Also, translational social neuroscience approaches, which aim at understanding the prevalent and fundamental deficits of interpersonal behavior associated with psychiatric disorders (e.g., Crespi and Badcock, 2008; Tost and Meyer-Lindenberg, 2012) and at improving therapeutic interventions by means of neuroimaging (e.g., Linden, 2006) may be impeded by a lack of ecological validity (cf. Insel, 2010). In particular and despite its obvious merits, much of the current work on socio-cognitive aberrations in clinical populations may have over-emphasized explicit forms of social cognition, e.g., the conscious effort to think about another person’s mental states, their neural correlates and their relationship to socially (in-) adequate behavior (cf. Schilbach et al., 2013). There is, however, mounting evidence that interpersonal difficulties might be more closely linked to impairments of social *interaction*, i.e., abilities and skills to unreflectively and yet successfully deal with others under the time constraints of an ongoing social exchange, rather than impairments of social *observation*, which are less pronounced in many clinical groups (Klin et al., 2003; Senju et al., 2009; Schilbach et al., 2012; Rietveld et al., 2013). This realization may also be of particular importance for psychotherapeutic interventions, which, in part, are beginning to focus more on the procedural nature of and skills associated with social interaction (e.g., McCullough et al., 2011).

An important avenue for future research in social neuroscience, therefore, consists in systematically investigating the neural basis of active participation in real-time social interaction. This endeavor has been described as the development towards a “second-person neuroscience”, which -according to some—promises to provide new insights into how activity in large-scale neural networks is modulated by social interactions (cf. Heyes, 2010, 2013; Gallese, 2013; Schilbach et al., 2013). Furthermore, the second-person view may hold some potential to reconcile current controversy between polarized versions of interactionist (Di Paolo and De Jaegher, 2012) and individualistic (Gallotti and Frith, 2013) approaches of social cognition by providing an account of how cognitive functions develop in the context and by virtue of interaction with others (Timmermans et al., 2013). Importantly, this is not meant to rule out the existence of an “observation” mode of social cognition as we can certainly infer things about others when we are observing them and make use of our ability to simulate others’ mental states as well (e.g., Waytz and Mitchell, 2011; Paulus et al., 2013), but rather to stress that it is the emergence of the capacity for reasoning about people, for inference and simulation that needs to be investigated and understood as resulting from our prior ability to interact with them and not how social actions and interactions emerge from our capacity to represent knowledge about others.

In essence, the second-person approach suggests that social cognition is fundamentally different when we are in interaction with others rather than merely observing them, thereby pointing towards the importance of experiencing and interacting with others as our primary ways of knowing them (Schilbach et al., 2013): on this view, firstly, awareness of other minds is thought to hinge upon emotional engagement and a responsiveness to another person’s states or actions as compared to a detached observer’s attitude, which does not include such responding (cf. Reddy, 2003). Here, emotional responses are thought to constitute an important way of perceiving and integrating the state of the other by way of experiencing one’s own bodily responses to her (e.g., Schilbach et al., 2008). Secondly, the process of social interaction is seen as a key constituent of grasping other minds. Social interactions are characterized by reciprocal relations with the perception of socially relevant information prompting (re-) actions, which are themselves reacted to. In social interaction, it has been suggested, we rely upon our practical “know-how” in dealing with others (Klin et al., 2003). Here, interaction and feedback from the other are not only a way of gathering “data” about the other person, but—in part—one’s knowledge of the other may reside in the interaction dynamics between the agents (e.g., Froese et al., 2014). Compatible with a Wittgensteinian perspective, this account also suggests that psychological concepts gain meaning by how they are used in everyday life encounters rather than being reliant on ontologically private states (Wittgenstein, 1953/2001). From this perspective, spectator theories of social cognition incorrectly assume that social cognition is grounded in inferences about others’ minds. Rather social cognition occurs within and is motivated by social interaction (Moore, 2013).

Consequently, a most important objective for a “second-person neuroscience” will have to be to shed new light onto the question of how particular cognitive functions actually are realized in particular areas of the brain of an interactor (or the brains of interactors) and continue to be modulated by participation in social interaction (cf. Westermann et al., 2007; Heyes, 2013). By aiming for this goal, this line of research could also close in on another hugely important topic in developmental psychology and social neuroscience, namely the relationship between the neural correlates of social interaction and the neural correlates of social observation. Developmental psychology clearly suggests a primacy of social interaction as the motor for developmental change, but also highlights the importance of social observation skills for learning. Here, an important suggestion is that the implicit processes relevant for participation in real-time social interaction with others may constitute our primary way of knowing them (Reddy, 2008) and that more explicit forms of self- and other-related knowledge may be accrued following and resulting from such interactions (e.g., Timmermans et al., 2012, 2013). Based on this assumed trajectory, one could postulate that neural networks relevant for social cognition are ontogenetically trained during social interactions, whereas later developing competencies of more detached, observational and finally reflective social cognition might be a result of reactivating the networks forged during social interactions. This suggestion, in fact, links up neatly with theories about the functional structure of the brain, which take the “re-use” of neural circuitry for various cognitive purposes to be a central organizational principle (e.g., Anderson, 2010). According to these theories, it is common for neural circuits to be established for one purpose and then to be put to different uses. Crucially, the relevant changes in neural network architecture could be brought about by the increasingly complex interactive social contexts, which create the possibility and need to communicate with respect to an external, third object or person (cf. Carpendale and Lewis, 2004). Furthermore, the involvement of reward signals during an ongoing social interaction might also be relevant (Guionnet et al., 2012; Baez-Mendoza and Schultz, 2013). Yet, to the best of my knowledge, no neuroscientific evidence exists to directly address these questions, despite the potentially far-reaching conceptual and clinical implications. In the following, I will, therefore, present some ideas for future research and study designs, which could help to investigate the neural correlates of social interaction and social observation as well as their putatively close relationship.

## Ideas for future research

### Investigating the neurobiological correlates of social interaction and observation

With regard to the neuroscientific question of whether or not similarities and/or differences between interaction-based and observation-based neural networks can be detected, an important challenge consists in finding neuroimaging-compatible experimental tasks, which allow for active participation in an ecologically valid way. To this end, we have developed a technique that uses eyetracking data obtained from participants inside the MR scanner to make a virtual character’s gaze behavior responsive to the participant’s gaze in real-time (Wilms et al., 2010). This setup, therefore, allows participants to experience their own eye-movements to have an effect on the gaze behavior of a (virtual) other, similar to how this may occur in real-life situations. This “interactive eyetracking” setup has been successfully used to perform fMRI studies, in which participants interacted with a virtual agent while undergoing neuroimaging. During one particular study, the gaze-based interaction included looking at objects “together” with the agent, thereby engaging in what is commonly described as “joint attention”, i.e., interpersonally shared visual attention, or looking at objects other than the one the virtual agent was looking at (Schilbach et al., 2010). Results of a univariate neuroimaging analysis demonstrated a differential increase of neural activity in brain regions that are commonly ascribed to the MENT during joint attention (i.e., looking at the same objects as compared to looking at different objects), such as dorsomedial prefrontal cortex and the precuneus, irrespective of whether or not joint attention was initiated by the participant or the virtual other. Looking at an object different from the one looked at by the virtual other led to the recruitment of a fronto-parietal network. In spite of the participants fixating objects in all experimental conditions, the underlying brain activity, therefore, was markedly different depending upon whether or not the participant was doing this “together” with the virtual other or not. Interestingly, activation differences in the MENT were observed even though participants were given no instruction to think about the mental states of the other. In fact, participants did not have any reason to do so. In the instructions they had been told that the virtual character seen on screen was controlled by another human participant, who was given instructions to behave in a certain way. These findings, therefore, contradict the idea that activity changes of the MENT are necessarily associated with reflective or explicit social cognition, but rather demonstrate that activity changes in this network can also be brought about by participation in reciprocal social interaction. This automatic activation of the MENT during joint attention with another agent could, therefore, be taken to suggest that activity in the MENT can be shaped and modulated during gaze-based social interaction. Furthermore, the results demonstrated a significant difference in the neural processes of joint attention that were dependent upon joint attention being self- as compared to other-initiated: here, a differential effect was found in the nucleus accumbens bilaterally, which is known to be an important component of reward-based neurocircuitry (e.g., Peters and Büchel, 2009). Also, activity change in this region was positively correlated with a pleasantness rating that subjects were asked to provide after the scan and during which they indicated that they enjoyed watching the objects more “together” with the virtual agent.

**Figure 1:**
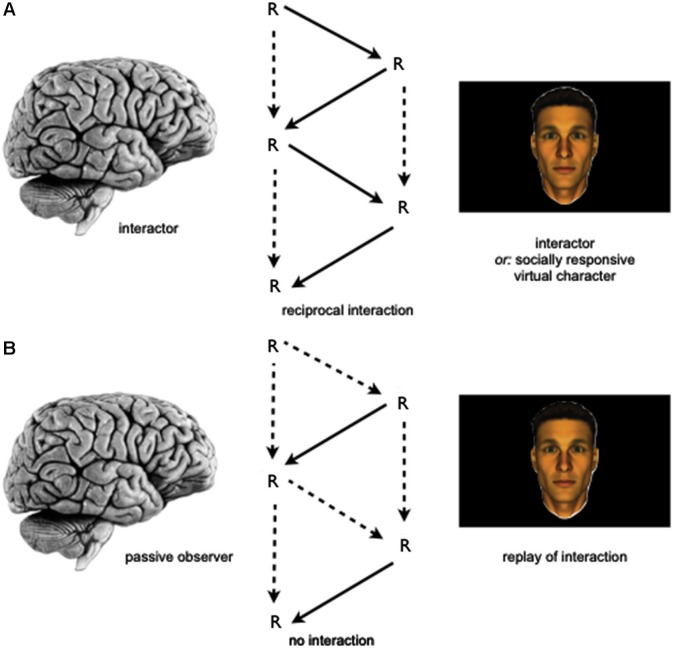
**Schematic depiction of an empirical approach to investigate the relationship of the neural correlates of social interaction and observation. (A)** Study participant (*left*) is involved in a gaze-based social interaction task with either a socially responsive virtual character (via “interactive eyetracking”, see Section Investigating the Neurobiological Correlates of Social Interaction and Observation) or an agent controlled by another human participant (via “dual eyetracking”, see Section Tackling the “Two-Brain Challenge”) while undergoing neuroimaging. During the interaction the task-relevant gaze behavior of both participants is being recorded. **(B)** In a separate group of participants, replays of task-relevant gaze behavior from the social interaction task are presented to a participant, who is asked to perform the same task, but rely upon the gaze behavior shown to him on the video screen. Here, again the participant’s brain (*left*) is investigated by means of neuroimaging to allow (e.g.,) for a group-based comparison between the neural correlates of social interaction and observation. *Legend: R: (re-) actions performed by agents. Dotted lines in schematic depiction of interaction contingencies indicate the absence or relatively decreased influence of actions on oneself (vertical arrows) or the other (oblique arrows), while solid lines indicate the presence of such influences. The temporal sequence of the social interaction is shown from top to bottom.*

Future research could make use of these interaction-based paradigms to compare brain activity changes during gaze-based interaction to activity changes during social observation (Figure 1). This could, for instance, be realized by providing a replay of a virtual character’s gaze behavior to participants as obtained during an interaction task to another group of participants undergoing neuroimaging. In the latter group, participants’ gaze behavior will, therefore, not produce any behavioral changes in the virtual other while using identical stimulus material, thereby eliminating the interaction contingency (see arrows, Figure 1). Furthermore, future research could also help to address differences between more structured, turn-taking types of interactions as compared to fully dynamic encounters and could focus in greater detail on effects of congruency, i.e., whether or not interactors perform similar, different or complementary actions. The latter distinction might also help to better understand the putative difference between social interaction and observation as those could be related to specific action tendencies.

Another extension of this line of research could consist in combining gaze-based interaction tasks with subsequently presented or interleaved tasks that measure pro-social tendencies and behavior, self-perception, social decision-making and advice-taking, in-group and out-group biases and gaze cueing (as compared to non-social cueing) effects (cf. Engemann et al., 2012). By doing so, the effects of social interaction on how we view ourselves and others and how we behave towards them could be examined. Also, the effects of participation in social interaction as compared to the effects of social observation on how we view the worlds and the decisions we make in it could be studied. Acquiring behavioral and neuroimaging measures both during the interaction task and the subsequently presented tasks would allow to investigate the modulatory impact of social interaction both on a behavioral and neural level as well as to use behavioral and neural markers obtained during the interaction as a predictor for performance and psychophysiological change during the subsequently presented tasks.

### Investigating the relationship of neurobiological correlates of social interaction and observation

As outlined above, another important question for the field of social neuroscience consists in asking not only how neural networks may be differentially engaged during real-time social interaction, but also whether and how these networks may also be involved in social observation and explicit social cognition. In this respect, it appears particularly relevant that an argument based on mere topography might not suffice. As discussed above, already today evidence exists to demonstrate that participation in gaze-based social interaction (e.g., looking at the same object together with another person in the absence of a necessity to think about the mental states of that other person) can lead to a differential increase of neural activity in the MENT (Schilbach et al., 2010) while the same brain regions are also activated during Theory of Mind-like tasks, which require explicitly thinking about another person’s mental states (e.g., Vogeley et al., 2001). Therefore, the investigation of the relationship between the neural correlates of social interaction and social observation will also have to take into account *how* the brain represents the social world rather than (exclusively) *where* in the brain the social world is represented. In this respect, machine learning approaches might prove to be particularly helpful as they allow to go beyond a mere mapping cognitive functions to brain regions based on differences in activation levels, but are sensitive to activity pattern differences (e.g., Kriegeskorte et al., 2006).

A particularly pertinent example of the putative relationship between a cognitive phenomenon that first becomes manifest as part of “online” social interaction, but is known to constitute a precursor and may, thus, turn into more explicit, “offline” social cognition is the phenomenon of “joint attention”. As indicated above, “joint attention” is an interactively constituted phenomenon that occurs when two agents coordinate their visual attention (e.g., Schilbach et al., 2010). Furthermore, joint attention constitutes a developmental milestone, which is known to precede other social cognitive abilities (Charman et al., 2000), such as the ability to explicitly represent others’ mental states (as assessed e.g., by means of the False Belief task; cf. Wimmer and Perner, 1983). Based on the idea of a psychological continuity between the phenomenon of gaze-based joint attention and other, later-developing explicit social cognitive competencies, one could, therefore, hypothesize that the neural correlates of participation in gaze-based joint attention are co-opted for other functions during ontogeny. It may, therefore, bear resemblance with the neural correlates of explicit social cognition.

**Figure 2:**
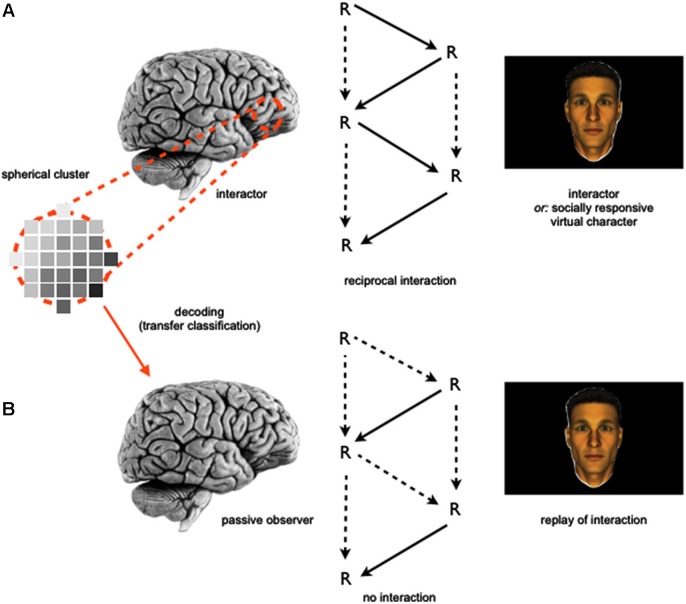
**Schematic depiction of an information-based neuroimaging approach to investigate the relationship of the neural correlates of social interaction and observation. (A)** A machine learning algorithm employing a searchlight approach (e.g., Kriegeskorte et al., 2006) is trained to separate brain states obtained during a social interaction task. **(B)** The machine learning algorithm is then used to classify a separately obtained data set, which includes brain states measured during social observation.

In order to address this question, participants could be involved in two-part fMRI studies similar to the rationale detailed above (Section Investigating the Neurobiological Correlates of Social Interaction and Observation): in a first part, subjects are asked to participate in a more implicit, gaze-based social interaction task, which e.g., could involve jointly attending to an object “together” with someone else (as compared to looking at an object different from the one the other is attending to). In a second part, participants will be asked to perform explicit social cognitive tasks, which e.g., could involve contemplating and naming the mental states of a human individual presented on screen (as compared to guessing her age) while also undergoing neuroimaging. In line with an approach adopted by Knops et al. (2009), machine learning algorithms could be employed to investigate this type of combined data set: here, a classifier could be trained that reliably separates brain states associated with distinguishable phases of the social interaction on the whole brain level (e.g., jointly attending vs. not jointly attending; leading the interaction vs. following the other). In a next step, it could then be tested whether the exact same classifier—without further training—would generalize to the second fMRI data set, based upon the idea that similar neural mechanisms and computations could underlie both participation in social interaction and social observation (Anderson, 2010; Schilbach et al., 2013). Based upon the idea that interaction-related network activity may be related or even a precursor for abilities of more detached social perception and observation, one would expect the social interaction-trained classifier to perform at a better-than-chance level on the social observation-associated data set (Figure 2). Thereby, this approach would allow to test whether the same neural mechanisms that are engaged during *online* social cognition are also put to use in situations of *offline* social cognition.

A similar type of experiment could be also be conducted to further investigate the MNS: in the context of controversy about the existence of this neural system in the human brain, activation-based findings have often been cited to in order to defend the view that we use our own motor system to perceive and understand the actions of others. While this type of neurofunctional isomorphism has been taken to suggest that the same neural mechanism is at work in both cases (interaction and observation), such a claim, one could argue, is beyond the scope of activation-based neuroimaging, because it only investigates whether the same brain regions show comparable levels of activation (i.e., where in the brain the cognitive process is localized), but not whether the same neural computations are relevant (i.e., how the brain realizes a certain function). Here, again multivariate or machine learning investigations could help to shed new light onto this question (cf. Etzel et al., 2008).

### Tackling the “two-brain challenge”

**Figure 3:**
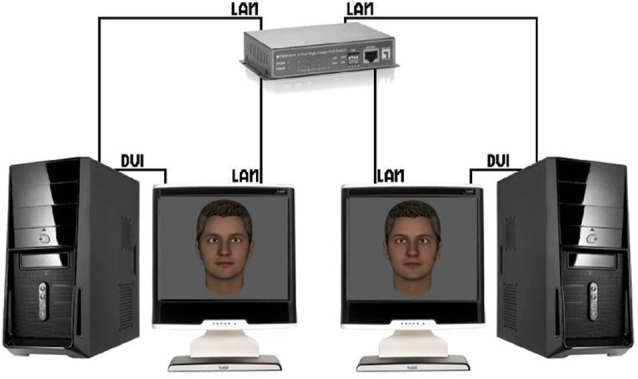
**Dual eyetracking setup.** Two eyetracking devices are linked via a local area network (LAN), which allows to simultaneously measure two study participants engaged in a mediated gaze-based interaction (each participant is represented by a virtual character for the respective other). *Taken from: Barisic et al. (2013).*

As outlined above, a large portion of previous work in social neuroscience has studied behavior and brain activity of individuals in isolation or as passive by-standers of social interactions (Schilbach et al., 2013). Recent developments towards a truly social neuroscience have, therefore, tried to establish what the next important steps of the field could be. One suggestion has been that the development of adequate experimental paradigms to investigate real-time social interactions will be of key importance (Wilms et al., 2010; Pfeiffer et al., 2013b). Useful paradigms should be ecologically valid, interactive and emotionally engaging while at the same time offering a high degree of experimental control. In addition to this, steps should be taken to go beyond testing individual brains, even though testing the brain of one person who is immersed in a life-like, real-time social interaction would already constitute a step forward (see Schilbach et al., 2013 for the difference between *second*-person and *two*-person neuroscience). An important challenge of two- (or multiple) person setups, however, consists in finding adequate and useful behavioral markers to quantify interpersonal aspects of the social interaction (cf. Konvalinka and Roepstorff, 2012). Here, a recent technical development in our group—inspired by previous work, which was successful in quantifying processes of interpersonal gaze coupling (e.g., Richardson et al., 2007; Jermann et al., 2010)—has involved linking up two eyetrackers via a local area network to form a “dual eyetracking” system (Barisic et al., 2013; Pfeiffer et al., 2013b), which enables real-time gaze-based social interactions of two individuals represented by virtual characters (Figure 3). In this setup, the gaze of two participants can be tracked continuously during the performance of joint tasks (e.g., two-person perceptual decision-making tasks). Apart from individual task performance (indicated by e.g., button presses), the eyetracking data from both participants can be analyzed to generate a measure or behavioral marker of the degree of interpersonal coupling (e.g., by assessing gaze recurrence; Anderson et al., 2013). Such a continuous measure of interpersonal coordination could be related to task performance in future studies, but also to the subjective confidence of participants during decisions. Furthermore, this trial-by-trial behavioral marker of interpersonal coordination could be used in future neuroimaging studies that employ the dual eyetracking setup to investigate its neural correlates in either one, or both of the brains of the two interactors.

With regard to the latter case of attempting to investigate the neural correlates of social interaction in two interactors’ brains at the same time, it has been suggested by Konvalinka and Roepstorff (2012) that machine learning could be a particularly powerful tool: in particular, such algorithms could help to elucidate whether in certain situations of an ongoing social encounter data from two brains is better at predicting social behavior than when the two brains are considered in isolation, thereby tackling the so-called “two-brain challenge”. On the other hand, more conventional analysis of neuroimaging data (e.g., univariate approaches as part of a general linear model) could also still be viable: using the above described dual eyetracking setup, for instance, a behavioral marker of interpersonal coupling could be used as a parametric regressor to investigate whether activity changes in certain brain regions (in one or both brains) is correlated with the extent of behavioral interpersonal coupling.

## Conclusions

In this Hypothesis and Theory Article, I have pointed towards current challenges and open questions in the burgeoning field of social neuroscience. In my view, these challenges—at least in part—consist in moving this field of research towards the investigation of the neurobiological correlates of real-time social interactions as the neural mechanisms, which “facilitate or inhibit social interaction have been difficult to assess” (Insel, 2010). Here, a variety of approaches might be useful. In the present article, I have focused on the potential of developing innovative experimental paradigms to investigate social interaction in a more ecologically valid and yet controllable fashion. The resulting, much needed investigations of real-time social interaction may initially involve the investigation of an individual brain in ongoing social interaction, which would already constitute an important achievement in comparison to a large number of currently available studies that focus on social observation. But future research should also turn towards the inclusion of two interacting minds (and possibly two brains; e.g., Tognoli et al., 2007; Dumas et al., 2010, 2012; Saito et al., 2010), even though this raises difficult issues with regard to an adequate quantification of interpersonal processes. Elucidating the neural correlates of social interaction will be a significant achievement in its own right, but it will also open up another avenue of research that deals with the investigation of similarities and differences in the neural correlates of social interaction and observation. Based upon evidence from developmental psychology and psychiatry, which suggest a primacy of social interaction over observation and the idea that neural networks established during social interaction may be “re-used” during observation, future research should focus on testing the exact nature of the relationship between the neural correlates of social interaction and observation. Combining and comparing the results of interaction-based paradigms with tasks that require social observation via the application of different tools for analysis is likely to provide new insights into this important relationship of *online* and *offline* social cognition.

## Conflict of interest statement

The authors declare that the research was conducted in the absence of any commercial or financial relationships that could be construed as a potential conflict of interest.
